# School-Based Physical Activity Intervention: A Qualitative Process Evaluation of a Feasibility Trial in Yangzhou, China

**DOI:** 10.3390/ijerph19021021

**Published:** 2022-01-17

**Authors:** Haiquan Wang, Yanxing Zhou, Holly Blake, Kaushik Chattopadhyay

**Affiliations:** 1 Division of Epidemiology and Public Health, School of Medicine, University of Nottingham, Nottingham NG5 1PB, UK; kaushik.chattopadhyay@nottingham.ac.uk; 2 The Nottingham Centre for Evidence-Based Healthcare: A Joanna Briggs Institute Centre of Excellence, University of Nottingham, Nottingham NG5 1PB, UK; 3 School of Humanities and Social Science, University of Science and Technology Beijing, Beijing 100083, China; zhouyanxing919@163.com; 4 School of Health Sciences, Faculty of Medicine and Health Sciences, University of Nottingham, Nottingham NG7 2HA, UK; holly.blake@nottingham.ac.uk; 5 NIHR Nottingham Biomedical Research Centre, Nottingham NG7 2UH, UK

**Keywords:** physical activity, qualitative study, process evaluation, interview, children, China

## Abstract

Background: There is an urgent need for children in China to increase their physical activity levels. We first developed a 16-week school-based behavior change intervention based on the Behavior Change Wheel and Theoretical Domains Framework. We then conducted a cluster feasibility non-randomized controlled trial (RCT) among children in Yangzhou, China. Aim: This qualitative process evaluation was embedded within the cluster feasibility non-RCT and aimed to: (1) explore the experiences and perceptions of participants and providers in the intervention and trial; and (2) generate recommendations to inform a future intervention and full-scale cluster RCT. Methods: A qualitative study, using semi-structured interviews with trial participants (*n* = 20 children: 10 intervention, 10 control), parents (*n* = 20), and health education providers (*n* = 2), was conducted in two public schools in Yangzhou, China. Interviews were audio-recorded, transcribed, and translated verbatim from Mandarin to English. Data were analyzed using thematic analysis. Results: Findings believed to reflect experiences and perceptions of participants in the intervention and the trial are presented as eight major themes: (1) perceived high efficacy of the intervention components to help children become more active, (2) appreciation of the intervention features, (3) factors that facilitated or impeded intervention attendance and delivery, (4) positive experiences and feelings gained through the data collection process, (5) satisfaction regarding the organization and implementation of the trial, (6) influences of personal beliefs and emotional responses to the trial, (7) social influences on participatory decision-making, and (8) key barriers to consider regarding the recruitment of participants. Conclusions: The intervention and trial methods were acceptable to children, parents, and health education providers. School-based behavior change intervention was perceived to be a useful approach to increase physical activity in children aged 10–12 years in China. However, there were barriers to intervention delivery and engagement that should be considered when designing a future cluster RCT to assess the intervention efficacy.

## 1. Introduction

Although regular physical activity is essential for physical and mental health [1,2,3], evidence shows that 81% of school-aged children are physically inactive globally [4]. Chinese physical activity guidelines recommend that children aged 5 to 17 years should engage in at least 60 min of moderate-to-vigorous physical activity (MVPA) per day and reduce their sedentary time [5]. However, over 84% of children do not meet the recommended physical activity level in China [4]. Children’s physical activity starts to decline at 10–12 years of age [6,7], which indicates a need for interventions at this early stage of life [8]. The research focused on physical activity promotion in children has been growing in the past two decades in China, and multiple studies have reported the positive outcomes associated with physical activity behaviors and weight management [9,10,11,12]. However, a consistent decline in physical activity time for children in China has been reported [6]. Several studies have revealed a decline in children’s physical fitness and health (e.g., strength, endurance) and an increase in the prevalence of overweight and obesity among children in China [13,14]. In schools, health (physical) education and structured exercise programs are available and delivered to the children orally and/or in written format. Structured exercise sessions are run to achieve the recommended intensity and duration of physical activity. The promotion of physical activity requires an understanding of the underlying influences on this behavior [15,16]. However, evidence suggests that these programs do not intervene in either the children’s intrinsic inclination towards physical activity or physical activity that occurs after school (e.g., weekends and holidays) [17]. In China, previous physical activity interventions for children lacked a theoretical basis for targeting the potential drivers of this behavior [18,19]. For instance, a systematic review on the effectiveness of physical activity programs has suggested that these programs in China are not developed and evaluated using systematic frameworks (such as the United Kingdom (UK) Medical Research Council (MRC) framework for developing and evaluating complex interventions) [20]. Around 80% of these physical activity programs are found to be of poor quality. The quality of interventions could be improved through consulting experts and by having patient and public involvement and engagement (PPIE) during intervention development, dissemination, and implementation; however, previous studies have often neglected or not reported these steps in the intervention development [21]. This could be due to the associated costs of PPIE, short timescales for delivery of interventions, not understanding the importance of PPIE steps, or having a tight word limit in peer-reviewed publications. We have addressed this issue, and our Joanna Briggs Institute (JBI) qualitative systematic review on barriers and facilitators to physical activity among ethnic Chinese children has synthesized four broad themes, namely, personal, sociocultural, environmental, and policy- and program-related factors [22,23].

Process evaluations are integral elements of complex behavior change interventions [15], which oversee the intervention delivery, the reach (number of people who receive the intervention), fidelity (the extent to which the intervention was delivered as planned), and implementation (how well the program was implemented) [24]. However, process evaluation has often been neglected or not adequately reported in previous physical activity programs for children in China [25]. This may undermine our understanding of participants’ experiences and perceptions of intervention delivery and participation in trials. Exploring the experiences and perceptions of those involved is essential for successful recruitment and retention in intervention trials, and to better understand what makes acceptable and effective intervention. The UK MRC framework for developing and evaluating complex interventions recommends a feasibility and piloting phase after an intervention has been developed [15]. At this stage, process evaluation can have a vital role in understanding the feasibility of the intervention and optimizing its design and evaluation. Moore and colleagues suggested that process evaluation can also be used for formative purposes to test the feasibility of intervention elements in a pilot prior to full implementation and to make implementation adjustments to ensure high dose, fidelity, and acceptance [24]. Depending on the stage of intervention development and implementation, researchers could use different components of process evaluation. Process evaluations are helpful during the feasibility evaluation of behavioral interventions as they can capture information on how the intervention may work in a trial setting as well as gather feedback from participants, intervention deliverers, and key stakeholders on how the intervention content could be improved to maximize the potential for behavior change [26]. Specifically, the information collected can be used to improve recruitment, increase follow-up, enhance attendance, and refine and improve the intervention design prior to conducting the full-powered definitive trial [26,27].

Behavior-change-theory-based interventions have shown positive effects on children’s health behaviors, such as physical activity [16]. However, evidence is still lacking regarding the feasibility and efficacy of theory-based interventions targeting physical activity of children in China, or the research processes used to evaluate them. We conducted a cluster feasibility non-randomized controlled trial (RCT) that examines the feasibility of delivering a 16-week behavior change intervention for increasing physical activity levels among children in Yangzhou, China [28]. Specifically, the study provided estimates of many important quantitative parameters (e.g., recruitment, follow-up, initial estimates of effects) needed to design the future cluster RCT. These are reported in detail elsewhere [28]. Briefly, the study indicated that the intervention may lead to improvement in children’s self-efficacy, enjoyment, and social support for physical activity. Although the cluster feasibility non-RCT analysis showed promising recruitment (100%), follow-up (100%), completion of data collection (100%), and intervention attendance (100%), more work is needed to explore intervention delivery and implementation, the influence of context, aspects of the intervention that were more or less successful, and how it could be improved to facilitate the desired behavior change. The aim of the study was to conduct a qualitative process evaluation to: (1) explore the experiences and perceptions of participants and providers in the intervention (i.e., intervention-specific questions for participants and providers who were in the intervention group only) and trial (i.e., feasibility-trial-specific questions for participants and providers who were in both intervention and control groups); and (2) make recommendations to inform the design of a future intervention and full-scale cluster RCT. The study was conducted in the context of our cluster feasibility non-RCT which examined the feasibility and acceptability of undertaking main cluster RCT with participants and providers in China [28].

## 2. Methods

### 2.1. Study Design, Location, Period, and Reporting

This qualitative study was conducted in two public schools in Yangzhou, China [28]. Participants in one school received the intervention in addition to their usual physical activities. Participants in the other school continued with their usual physical activities. In this qualitative study, semi-structured interviews were conducted with children, their parents, and health education teachers at the end of the intervention (from September 2020 to October 2020, by prior appointment). Reporting of this study was guided by the Consolidated Criteria for Reporting Qualitative Research (COREQ-32) guidelines (see Supplementary File S1) [29].

### 2.2. The Behavior Change Intervention

We used the Behavior Change Wheel (BCW), Theoretical Domains Framework (TDF), and information gathered through PPIE activities to systematically develop a school-based behavior change intervention [16]. The intervention was developed with the aim of encouraging children to be more active during the school day and increasing their overall physical activity levels (including leisure-time physical activity after school and at weekends). Children’s physical activity is recognized as being unstructured and intermittent, which may not be encompassed by traditional approaches (e.g., structured exercise programs). Instead, redesign of the physical activity environment may help to provide children with more opportunities for being active, in addition to the provision of quality and mandated physical education. In our intervention development phase, our behavioral diagnosis revealed that physical activity engagement among children in China was largely determined by TDF domains of environmental context and resources, social influences, beliefs about consequences, memory, attention, and decision process, emotion, and belief about capabilities [30]. Therefore, in the development of our intervention, we chose to focus on active behavioral change (i.e., promoting at least 60 min of MVPA per day) in children, rather than focusing on specific types of physical activity. The intervention development is reported in detail elsewhere [30]. Briefly, the intervention consisted of three components: (a) health education (physical education), (b) family involvement, and (c) school environment support (see Supplementary File S2). Health education was achieved by imparting knowledge to children via four group activity sessions and the provision of printed materials (e.g., activity diary). Examples of key features include: (a) provision of information about health consequences/emotional benefits to increase understanding, (b) demonstration of the physical activity domains to act as persuasion, (c) emphasis on previous successful experience and personal capacities to encourage children to perform the activity voluntarily, and (d) encouragement of goal setting and action planning for physical activity participation. Family involvement was achieved by: (a) an online education session and a physical activity booklet delivered to parents that elicited their positive feelings and impulses relating to physical activity promotion among their children; and (b) provision of emotional social support (e.g., buddying up with children for physical activity) and practical social support (e.g., encouraging parents to prepare sports gear for their children). School environment support was achieved by organizational changes, including: (a) restructuring aspects of the physical environment (e.g., provision of physical activity poster, sports equipment, and pedometer) to increase children’s initiatives for performing physical activity; and (b) provision of verbal encouragement or material reward (e.g., stickers).

### 2.3. Study Participants and Recruitment

All children, parents, and teachers who participated in the cluster feasibility non-RCT were eligible to take part in a semi-structured interview. Particularly, the interviews for children and their parents were conducted in dyads (pairs). Child-parent dyads were purposively selected and contacted by an independent teacher. Two health education teachers from the intervention (*n* = 1) and control school (*n* = 1) were contacted directly by the study team for independent interviews. Children aged 10 to 12 years with verbal assent and parents’ consent were interviewed. Parents and health education teachers with verbal assent and informed consent were interviewed.

### 2.4. Sample Size

In the cluster feasibility non-RCT, we recruited a total of 64 child-parent dyads (32 per study group). Interviews aimed to achieve data saturation to the point where no new or relevant data were discovered in the analysis [31]. Evidence suggests that the majority of themes in a homogenous sample tend to be captured in around six to seven interviews (i.e., six interviews to reach 80% saturation) [32]. The researchers believed that data saturation was reached after the 10th interview among child-parent dyads in both intervention and control groups, when no new information was arising from the discussions. However, in the case of providers, only two health education teachers were part of the trial, i.e., one in the intervention group and one in the control group. The characteristics of the interviewees are summarized in Table 1.

### 2.5. Interview Guides

This study followed the structured process evaluation framework proposed by Reelick and colleagues [27]. The framework was chosen for the current study for its systematic and comprehensive guidance for process evaluations at the feasibility stage of development. The framework evaluates three main components of intervention and research processes: (a) the success rate of recruitment and quality of the study population; (b) the process of data collection; and (c) the quality of implementation of the intervention. Each process component can be assessed by several measures and multiple variables (Table 2). As such, three semi-structured interview guides (see Supplementary File S3) were adapted, one each for children, their parents, and health education teachers from similar feasibility studies [33,34]. These were then translated from English into Mandarin, and the translation was checked for accuracy by a bilingual researcher (HW). The three interview guides are largely the same for general questions that explored participants’ and providers’ experiences and perceptions in the intervention (e.g., experiences and perceptions of participating in the education sessions, perceptions of intervention frequency and timing, and attitudes toward the intervention providers; intervention-specific questions were asked in the intervention group only) and feasibility trial (e.g., recruitment, understanding of the information sheet, and data collection; feasibility-trial-specific questions were asked in both intervention and control groups). Meanwhile, the interviews included specific questions for different categories of participants (e.g., questions relating to the use of the activity diary and physical activity booklet for children and parents, respectively).

### 2.6. Interview Procedures, Transcription, and Translation

Prior to commencing the interviews, the purpose and procedures of the interview were explained. Participants (i.e., children, parents) and providers (i.e., health education teachers) were assured that their participation in the interviews was entirely voluntary, and they could stop the interview at any time during the process of interviews. The interviewer (HW) was a researcher (male) and trained in qualitative research methods. Interviewees had no prior relationship with the interviewer. All interviews were conducted in Mandarin using the telephone; interviews lasted 20 min on average (i.e., from 15–25 min). Face-to-face interviews were originally planned but were rescheduled to take place by telephone due to the social-distancing regulations of schools, put in place due to the COVID-19 pandemic at the time of the study. During the interviews, the interviewer used open-ended questions and constantly reminded interviewees that there were no right or wrong answers to help prompt free speech. The researcher made notes about the interview (e.g., insights about the interview, emerging points of interest), which helped in coding and generation of themes. With consent, interviews were audio-recorded. Transcripts were imported into QSR International’s NVivo version 12 software to enable data storage and organization for analysis [35]. Transcripts were not returned to the participants and health education providers, but efforts were made to minimize transcription errors and omissions. Specifically, encrypted files were then transferred to an external professional company for transcription (verbatim) and translation (from Mandarin into English) after signing the nondisclosure agreement. Moreover, two researchers (HW/YZ) constantly checked the transcripts provided by the transcriptionist against the audio recording to further rule out the possibility of errors and missing data.

### 2.7. Ethics

Ethical approval was obtained from the Faculty of Medicine and Health Sciences Research Ethics Committee, University of Nottingham, UK (255-1902).

### 2.8. Data Analysis

Transcripts were analyzed using thematic analysis [36,37], using both inductive and deductive approaches. Data analysis began with an inductive approach (i.e., codes arose purely from the data) to ensure important aspects of the data were not missed. In addition, codes could also be theory-derived (i.e., codes arose through a deductive approach), which allowed the researcher to be sensitive to existing knowledge that may arise in the data. For example, initial codes of “fun”, “personal interests in physical activity”, and “curiosity about physical activity knowledge” were deductive, as evidence has suggested that internal motivation could be a facilitator to children’s physical activity participation. The analysis was iterative rather than a rigid linear process, and all these codes were refined during analysis as the data emerged (inductive). All transcripts were rechecked, read, and reread by the two researchers (HW/YZ) independently to ensure credibility. The initial codes were developed through discussion between the two researchers, and applied initially to a small number of transcripts, enabling further discussion and iteration of the coding. Transcripts were independently coded by the two researchers, and the codes were discussed between them if any discrepancies were found in order to reach a consensus. The reviewers analyzed and coded the children’s transcript first, followed by that of parents and teachers. Similar codes from children, parents, and teachers were then collated together, which led to the emergence of themes and subthemes within each overarching category. In addition, specific codes relating to children, parents, or teachers were collated and grouped on the basis of similarity in meaning of quotations (e.g., codes related to the activity diary for children or physical activity booklet for parents). The identification of specific themes and subthemes relating to children, parents, or teachers was based on the similarity in meaning of the specific codes. Efforts were made to minimize bias by (a) involving researchers (HB, KC) who had not conducted interviews in analytic decisions relating to data interpretation, (b) applying reflexivity (HW) and examining reflective accounts (HW, YZ) during analysis processes, and (c) involving a researcher (YZ) in the analysis of data who was not involved in intervention delivery or cluster feasibility non-RCT design. Participants and providers did not provide feedback on the findings.

## 3. Results

Findings believed to reflect participants’ and providers’ experiences and perceptions in the intervention and trial are presented as eight themes. Within these eight themes, there were 32 subthemes. Themes, subthemes, and verbatim quotes are presented in Table 3 and Table 4.

### 3.1. Topic 1: Participants’ and Providers’ Experiences and Perceptions of Participating in the Intervention

Theme 1: Perceived high efficacy of the intervention components to help children become more active

This theme explored whether the intervention components were perceived by participants and providers to be suitable and beneficial to help children become physically active in their daily routine. Five subthemes were identified in regard to which components of the intervention helped children to keep active.

(1.1) Activity diary plays an active role in helping children review behavior goals

The activity diary received positive evaluations from children. All children could appreciate the information about physical activity and its benefits. Some felt that the physical activity plans and step logs in the activity diary reinforced their motivation for performing physical activity, and this encouraged them to continue being active (see Table 3, quote 1.1.1 to 1.1.3).

(1.2) Positive impact of the group activity sessions on physical activity beliefs

Both children and parents deemed that the attendance of group activity sessions helped them in better understanding physical activity and spending more time actively engaging in physical activity. Moreover, a number of parents explicitly reported having observed their children becoming more physically active after attendance in the group activity sessions (see Table 3, quote 1.2.1 to 1.2.3). In the third group activity session, we facilitated a poster-making session to provide the children with opportunities to reflect on the knowledge they learned, and use the production of physical activity posters as a way of demonstrating and reinforcing their understanding of physical activity. Overall, children acknowledged the session was interesting and appreciated it as a “burden-free” way to promote self-motivation in physical activity (see Table 3, quote 1.2.4 to 1.2.5).

(1.3) Increased awareness of ways of doing physical activity

Parents frequently discussed the usefulness of the examples and illustrations given in the physical activity booklet. They also highlighted the benefits of using the physical activity booklet to help actively support physical activity participation in their children (see Table 3, quote 1.3.1 to 1.3.2).

(1.4) Increased actions of self-monitoring through the provision of pedometers

The pedometer was a popular component of the behavior change intervention for children. Children expressed that they actively used the pedometers to monitor and reflect back on their daily and weekly step count, which in turn further motivated them to increase their amount of walking (see Table 3, quote 1.4.1). Parents and teachers referred to the pedometers as providing “invisible” supervision that helped children to self-monitor their physical activity levels and establish a motivating element of “competition” between children for comparing their daily step counts (see Table 3, quote 1.4.2 to 1.4.4).

(1.5) Positive influences of sports equipment on children’s unstructured and intermittent physical activity pattern

It appears that both children and parents found the provision of sports equipment to facilitate children in actively performing physical activity at any time. They valued the convenience of having the sports equipment available, which could be accessed by children whenever they chose (see Table 3, quote 1.5.1 to 1.5.2).

Theme 2: Appreciation of the intervention features

In general, children and their parents found the intervention to be acceptable and they were particularly content with the organization and delivery of the intervention (e.g., in terms of materials provided and the venues) and the opportunity to be involved. Five subthemes emerged in regard to the feelings around acceptability of the intervention features.

(2.1) Understanding of intervention materials (i.e., activity diary, physical activity booklet)

Children and their parents perceived that the information and expressions in the written intervention materials (i.e., booklets, activity diary) were well-prepared, informative, and understandable to them (see Table 3, quote 2.1.1 to 2.1.2).

(2.2) Satisfaction with venues

Most children expressed high levels of satisfaction with the venue (e.g., using health education to deliver the session in the school setting). Children stated that the venue was convenient and easily accessible. However, one child expressed a preference for the session to be scheduled at other timeslots instead of taking up the health education session time. In addition, being able to interact with the study team face-to-face was also often reported to be an advantageous feature in the intervention delivery (see Table 3, quote 2.2.1 to 2.2.2). Parents discussed that having the parental session conducted remotely was perfect as they could arrange their time appropriately (see Table 3, quote 2.2.3).

(2.3) Researcher’s characteristics in delivering the intervention

Children and their parents spoke positively about the personality traits and professional characteristics of the researcher delivering the intervention. They described the researcher as motivating, encouraging, helpful, and the communication with the researcher went well during the delivery of group activity sessions (see Table 3, quote 2.3.1 to 2.3.2).

(2.4) Acceptability of the group activity sessions’ duration, frequency, and timings

All children felt that the duration of group activity sessions was appropriate. However, there were mixed feelings among children about the frequency of group activity sessions. For example, one child expressed an expectation for more sessions whereas another child reported a preference for fewer sessions to be held in one semester (see Table 3, quote 2.4.1 to 2.4.3). Similarly, parents reported different perceptions towards the duration and frequency of the group activity session for parents. The majority of parents found the duration and frequency to be appropriate and acceptable while some parents advised to reduce the duration of the session or introduce more sessions in a semester (see Table 3, quote 2.4.4 to 2.4.6).

(2.5) Social interaction and engagement

At the end of the intervention, a number of children and parents expressed their desire to be future ambassadors for the intervention and help to disseminate it to more peers and friends. Children and their parents also expressed their desire to share the experience and knowledge they had learned from the group activity sessions with others (see Table 3, quote 2.5.1 to 2.5.2).

Theme 3: Factors that facilitated or impeded intervention attendance, and delivery

This theme draws on participants’ experiences and perceptions of taking part in the intervention to derive potential facilitators and barriers to intervention attendance and delivery. Specifically, there were four subthemes raised by participants and providers regarding the factors that facilitated or impeded intervention attendance and delivery.

(3.1) Gaining support from schools and education departments

All participants gave a positive evaluation of the organization of the intervention. The variety of intervention components was considered to be appealing. However, parents suggested that implementing the intervention at a wider level would require understanding and cooperation from schools and education departments (see Table 3, quote 3.1.1).

(3.2) Provision of electronic materials

One parent suggested that an electronic version of intervention materials may be easier to keep and more accessible than a hard copy (paper version). In addition, both parents and teachers discussed ways of providing the information related to physical activity in an electronic format (e.g., audio or video clips as a resource) or on social media platforms (e.g., promotion via Twitter) (see Table 3, quote 3.2.1 to 3.2.3).

(3.3) More physical activity options

Although parents believed that the provision of sports equipment facilitated an increase in children’s physical activity levels, one parent suggested including further activity options to cover a wider range of physical activity categories (see Table 3, quote 3.3.1).

(3.4) More instruments for self-monitoring

The parents thought the pedometers provided to children were helpful for children’s self-monitoring of their daily physical activity levels. They expressed an expectation for user-friendly devices for their children to use to monitor their physical activity levels (see Table 3, quote 3.4.1 to 3.4.2).

### 3.2. Topic 2: Participants’ and Providers’ Experiences and Perceptions of Participating in the Trial

Theme 4: Positive experiences and feelings gained through data collection process

Participants and providers seemed to have a clear understanding of the aims of the research trial. They appreciated the information about physical activity for children and felt it was appropriate and beneficial for children to measure their own physical activity levels. Five subthemes emerged from the interviews which were grouped under the broader category of experiences and feelings gained through the data collection process.

(4.1) Understanding of the translated Mandarin version of information sheets

Participants and providers expressed a common view on the acceptability of the information sheets. Most participants found the information sheets and explanations to be informative and straightforward. Yet, one parent found the materials to be over-complicated and suggested the materials would be improved if “simplified”. All teachers believed the explanations in the information sheets were clear and provided sufficient study details to the participants (see Table 4, quote 4.1.1 to 4.1.6).

(4.2) Perceived “burden-free” completion of self-reported questionnaire

All children felt the questions listed in the questionnaires were very comprehensible to them, and this was seen to expand their knowledge about physical activity (e.g., types of activities and examples of how to do it). The children felt that they had no problems in completing the self-report questionnaire and as such, it was seen to impart a low level of burden (see Table 4, quote 4.2.1 to 4.2.2).

(4.3) Positive reflection and self-feedback gained from completing seven-day steps’ measurement

All children positively discussed the measurement of seven-day steps that they received on their pedometer. Children found the measurement interesting, and they felt motivated and satisfied by the visibility of their achievements on the device (i.e., steps they walked). Children also liked that the pedometer was portable (see Table 4, quote 4.3.1 to 4.3.2).

(4.4) Anthropometric measurement promotes self-monitoring behavior and active emotional response

Children considered the anthropometric measurement to be an acceptable component in our trial, which could help them to better understand their own body (see Table 3, quote 4.4.1 to 4.4.2). Moreover, children did not report any negative feelings or experiences during the delivery of the measurement.

(4.5) Satisfaction regarding the organization of data collection

Children reported that they enjoyed participating in the data collection in our trial. They appreciated the process of the data collection which was seen to be well organized and delivered. Some children talked about the implementation of data collection as convenient and not time-consuming, which led them to suggest that the data collection had no negative influence on their schedule at school (see Table 4, quote 4.5.1 to 4.5.2).

Theme 5: Satisfaction regarding the organization and implementation of the trial

There were two broad subthemes that emerged from the interviews related to participants’ and providers’ perceptions of participation in this trial. In general, these demonstrated that the main benefits of the involvement in the trial were the experience and feelings of enjoyment, and satisfaction gained from interacting with others.

(5.1) Satisfaction with the trial content, organization, and engagement

All children and parents perceived participation in the trial positively, and they reported that taking part was satisfactory and enjoyable. Specifically, the main emotional feeling of children was the enjoyment gained from engaging in the trial. Parents discussed feelings of encouragement and motivation from observing their children and how children enjoyed communicating and sharing the trial information with them (see Table 4, quote 5.1.1 to 5.1.4).

(5.2) Meaningful and unprecedented experience

All parents and teachers felt that participation in the trial was a beneficial and meaningful experience in children’s development. They felt that an important feature of the trial was the provision of a new approach for physical activity education for children, parents, and teachers. Teachers also found that participation in the trial could help cultivate children’s interest in physical activity and generate an in-depth understanding of physical activity (see Table 4, quote 5.2.1 to 5.2.4).

Theme 6: Influences of personal beliefs and emotional responses to the trial

Understanding what motivated people to participate in this trial may help with future engagement of those who may be less likely to take part in an intervention trial. In general, all participants and providers expressed high enthusiasm to participate in this trial. The facilitators for participation in the trial could be grouped into five subthemes.

(6.1) Personal motivation for doing physical activity

Children were happy to be approached by the study team and they believed that participation in the trial would provide a great opportunity for them to perform more physical activities and understand their physical activity level (see Table 4, quote 6.1.1 to 6.1.2). Parents and teachers stated that their desire to encourage children to engage in such a trial is primarily due to the dearth of physical activity programs available for children in China (see Table 4, quote 6.1.3 to 6.1.4).

(6.2) Curiosity about the trial content

Some children and parents were curious about the content after the enrolment in the trial and were extremely enthusiastic about doing physical activity (see Table 4, quote 6.2.1 to 6.2.4).

(6.3) Desire to advance physical activity knowledge

Some parents described being motivated by the opportunity to help towards advancing scientific knowledge about physical activity, and receiving information about ways to help children keep fit (see Table 4, quote 6.3.1 to 6.3.2).

(6.4) Perceived physical-activity-related benefits

Children valued the health benefits that could be gained from participating in physical activity and discussed being motivated to participate in the trial as this would help improve an individual’s well-being. Parents stated that participation in physical activity could act as a way to help relieve the academic pressure on their children and ultimately improve their health. Teachers stated that participation was beneficial to children’s personal development (see Table 4, quote 6.4.1 to 6.4.6).

(6.5) Using vivid expressions and appealing advertising materials

Some children suggested that the trial could trigger children’s interest in participation even more by using vivid language and cartoons. They believed that the combination of text and cartoon pictures would be beneficial for future recruitment of children into this sort of trial (see Table 4, quote 6.5.1).

Theme 7: Social influences on participatory decision-making

Participants’ and providers’ decision-making to participate in the trial was not determined by personal beliefs and emotional responses independently. However, the social influences derived from the interpersonal and school levels appeared to play a significant role in their decisions to take part. Three subthemes emerged in regard to the social influences on participation in the trial.

(7.1) Interpersonal influences by children and teachers

After initial contact and recruitment under the help of head teachers in two schools, children went on to spread the information to their parents, and parents were also informed by the teachers about the trial information. Parents stated that their participation in the trial was influenced by the teachers as they valued teachers’ suggestions and recommendations (see Table 4, quote 7.1.1 to 7.1.2).

(7.2) Preferred recruitment in the school context

Schools emerged as a preferable context for reaching children and their parents. It was also identified as a safe and formal approach to have parents’ consent for their child to take part in the trial—this appeared to be an important reason that children and parents expressed interest in participation (see Table 4, quote 7.2.1 to 7.2.5).

(7.3) Highly influenced by the curriculum that had been set up in schools

Parents valued the role of physical activity for children and believed that their participation would be facilitated if the schools gave more attention and importance to delivering physical activity in the school environment (see Table 4, quote 7.3.1).

Theme 8: Key barriers to consider regarding the recruitment of participants

Children and parents expressed interest in participating in the trial, but some of them discussed potential barriers that might impede their participation in a future trial. These barriers to recruitment in the trial could be grouped into three subthemes.

(8.1) Academic pressure faced by children

Children’s academic achievements are heavily emphasized in China and schools are usually evaluated based on academic performance. Therefore, children and parents reported that participation in the trial might be limited by academic commitments. They felt this was a barrier to recruiting participants into a future study as children in China spent the majority of their leisure time working on assignments (see Table 4, quote 8.1.1 to 8.1.4).

(8.2) Time constraints on parents

Although parents did acknowledge their positive role in supporting and helping the establishment of children’s physical activity behaviors, lack of time was a common reason for parents failing to take part in the trial, and this should be taken into account to accommodate parents’ schedule when conducting the future study (see Table 4, quote 8.2.2).

(8.3) Lack of emphasis on physical activity at parental, school, and education department levels

There was a general lack of emphasis on children’s physical activity among parents, schools, and the education department in China. Children expressed that their parents prioritized academic attainment ahead of physical activity. On the other hand, parents believed that schools and the education department would need to tackle a top-down approach to reevaluate the importance of physical activity in children (see Table 4, quote 8.3.1 to 8.3.2).

## 4. Discussion

To our knowledge, this is the first qualitative process evaluation of a physical activity intervention conducted with school children in China. Our study used a standardized framework to explore participants’ and providers’ experiences and perceptions with the intervention and feasibility trial. This could be further used to directly inform the design of the main cluster RCT.

All the intervention components were positively valued by children, their parents, and health education teachers. Children appreciated the tips on how to keep themselves physically active and enjoyed using the step-count log in the activity diary, which induced them to engage in physical activity behaviors and facilitated self-monitoring on steps, respectively [33]. Comparably, the importance and positive efficacy of providing physical-activity-related education materials for children has been suggested in substantial physical activity programs [9,10,33,38]. In our trial, the perceived benefits of the poster-making session included opportunities for children to self-reflect on their understanding of physical activity and stimulate their interests in physical activity [10]. This is consistent with published literature suggesting that children can gain a feeling of enjoyment in physical activity interventions which involve engaging and “fun” components [9,10]. Moreover, children’s personal interests, feeling of enjoyment, and positive attitudes towards physical activity gained from our poster-making session enhanced their desire to engage in physical activity [39]. As for parents, they appreciated the group activity session which was seen to be a particularly valuable opportunity for them to gain an insight into how children should engage in physical activity and how they could help their own child to be more physically active. The use of group activities to encourage social support networks among parents and parental engagement has been discussed in previous physical activity programs [10,33]. In agreement, it highlights the need for multicomponent physical activity programs to incorporate physical activity resources for families [40]. The use of environmental restructuring, self-monitoring, and goal-setting to motivate and engage children in participation in physical activity has been discussed and valued in systematic reviews around effective behavior change techniques to increase physical activity in children [41,42]. Furthermore, parents in our study suggested a desire for more objective tools (e.g., tracking devices, pedometers) that can provide children with instant and direct feedback on an individual’s physical activity level to help children achieve different milestones and accommodate personal goals. This insight supports our previous research suggesting that the provision of activity tracking tools may reduce attrition among intervention participants [23,30]. It is also in accord with an earlier study indicating that wearable technology (e.g., wristwatch, activity tracker) should be used for physical activity data collection to meet the technology-related expectations of children and parents [38]. These qualitative findings suggest that there was an increased frequency in children’s physical activity involvement through the intervention, which may contribute to an increase in participants’ overall physical activity levels per day. This also alludes to a potential dose-response relationship (i.e., children are more active if they engage more in the intervention); however, this would need to be explored further in a future full-powered definitive trial. Moreover, future research would need to explore the relationship between children’s engagement in the intervention, physical activity, and their academic performance [43].

Our results suggested that participants and providers were receptive and appreciative of both written intervention materials (i.e., activity diary, physical activity booklet) and verbal instructions (i.e., mutual communications between study team, parents, and teachers). Importantly, both parents and teachers suggested that the intervention materials may be best provided electronically, and could include videos (to model activity plans), links to websites, or official accounts for self-exploration of physical activity information among children and parents. These findings are in line with previous studies that suggest the interactive digital technology could help deliver health information to improve health outcomes [38,44]. Specifically, evidence shows that digital technology can improve communication between researchers and participants, improve participants’ knowledge, and encourage behavior change [44]. In agreement, these additional social and mobile elements may enhance intervention success, and there are a growing number of examples of such methodologies (i.e., mobile interventions) within the physical activity promotion literature [45,46,47]. In terms of the organization of the group activity sessions (i.e., venues, timing), we had a mixed reception to the duration and frequency of the group activity sessions. This was similar to other physical activity studies that included an education component whereby some participants found it appropriate (e.g., duration, frequency, location) and others did not [9,33]. Specifically, some parents suggested that the parental group activity session could be made more acceptable by reducing the duration of one session and instead having a greater number of separate sessions to help better engage the parents in the health education. This finding was consistent with our previous work in developing the school-based behavior change intervention, in which we found that parents’ time constraint is a critical issue, as their work commitment can lead to insufficient parental support and negatively affect the engagement of parents within the group activity session [23,30]. Together, these findings highlight the practical constraints to setting up and running group activity sessions for children and parents.

Overall, participants and providers perceived participation in the trial as a satisfactory and meaningful experience. In this trial, we used a participatory approach in the recruitment of children and their parents by engaging health education teachers as facilitators for the promotion of our trial within the schools. Despite the impact of the COVID-19 pandemic, our results indicate that participants would prefer recruitment via teachers, and suggest that this generates a feeling of safety and formality when approached in the school context. However, our findings differ from previous family-oriented physical activity studies which have reported challenges when recruiting families into intervention research within their pre-agreed timescale [48,49]. Specifically, a recent systematic review and Delphi survey investigating effective and resource-efficient strategies for recruiting families into health-promoting intervention research identified 49 eligible studies, of which only 33% of studies (i.e., *n* = 16) reported a target sample size [49]. Of these 16 studies, it found that only 62% recruited a sufficient number of participants. A subsequent Delphi survey of 107 experts showed that only 38% recruited their target sample size over a median of 12 weeks. In contrast, recruitment periods were extended in 33% of studies with a median extension of 20 weeks [49]. This highlights the importance of “context” in improving recruitment to research studies. For instance, our findings indicate that school is an appropriate place to recruit children and their parents in China [50], but this may or may not be transferable to other countries and regions due to differences in culture and expectations. Although we achieved high recruitment, future recruitment strategies may be enhanced further by incorporating vivid illustrations and user-friendly language to attract children to take part. Participants in future trials are likely to express interest if the study team promotes the importance of physical activity to participants and appropriately discusses the research processes and intervention content that participants will experience in the trial [22,23]. Comments from our participants suggest that persuading schools and health education departments about the importance of physical activity and inducing organizations to give more emphasis to physical activity promotion in the school setting would be required to facilitate the implementation of the future cluster RCT [10]. Related to data collection, our results indicate that our assessments were well accepted by children; this is also reflected by the high response rate in completion of data collection in our trial [28]. Therefore, the use of our data collection methods may remain similar in a future cluster RCT. Only one parent reported a lack of understanding of study materials, although this might be avoided in future with opportunity for face-to-face discussion about the trial processes which was not possible here due to the restrictions of the COVID-19 pandemic. We recommend that, in a future cluster RCT, expanding the mode of delivery of recruitment information (i.e., offering face-to-face, offline meetings as well as written materials) may maximize recruitment rate and participant satisfaction.

### Strength and Limitations

We acknowledged the impact of our preconceptions on the data collection and interpretation of data. However, the involvement of multiple researchers from different backgrounds (physical activity, behavior change, health psychology, sociology) in the study design, conduct, and analysis improves the credibility of our findings. The qualitative, thematic analysis methodology employed generated rich data, giving a more in-depth understanding of the experiences and perceptions of children, parents, and teachers. To ensure the reliability and validity of the research findings, the researchers were constantly involved in a reflexivity exercise about biases and their effects on decisions related to different phases of data collection and analysis. Participation in the study was voluntary. Thus, the study may have reached the children, parents, and teachers who had a more favorable perception of this intervention and trial, which could lead to over-reporting of positive experiences and perceptions and/or under-reporting of more negative experiences and perceptions. Although the quality of transcripts was ensured through external company transcription and member checking, there is a risk of incorrect details that are most likely only detectable by the interviewees since we did not return transcripts to participants and providers due to time limitations. However, we prepared the interview guides in advance and used reminders (i.e., informing participants and providers that there were no right or wrong answers) to ensure that we included all aspects of participants’ experiences and perceptions. The findings of this study were limited to the views of families recruited from one geographical region and parents who were employed. Future studies should explore intervention engagement in more diverse participant groups, including children with parents who are not employed, those with lower income, different age groups, and those living in rural or remote areas. Although evidence of long-term effects was beyond the scope of this study, key elements of acceptability were identified. Results demonstrated success of the intervention delivery, acceptable research methods and processes as perceived by the participants and providers, and supportive evidence for the short-term efficacy of the intervention in some outcomes. Further exploration of the intervention’s effectiveness and sustainability is required in the next stages of MRC framework for developing and evaluating complex interventions (i.e., evaluation phase and implementation phase, respectively).

## 5. Conclusions

The intervention and trial methods were acceptable to children, parents, and health education teachers. School-based behavior change intervention was perceived to be a useful approach to increase physical activity in children aged 10–12 years in China. Interview participants highlighted the increased frequency of children’s involvement in physical activity as a result of the intervention, which may lead to their achievement of 60 min of MVPA per day. However, there were barriers to intervention delivery and engagement that should be considered when designing a future cluster RCT to assess the intervention efficacy.

## Figures and Tables

**Table 1:** Participants’ and providers’ characteristics by group.

	Intervention	Control
Children, *n*	10	10
Age in years, (range, mean)	10–12, 11.2	10–12, 11.2
Gender		
Female, *n* (%)	5 (50%)	4 (40%)
Male, *n* (%)	5 (50%)	6 (60%)
Parents, *n*	10	10
Mother, *n* (%)	6 (60%)	8 (80%)
Father, *n* (%)	4 (40%)	2 (20%)
Education		
High school diploma or equivalent (0–12 years), *n* (%)	5 (50%)	8 (80%)
University or equivalent (>12 years), *n* (%)	5 (50%)	2 (20%)
Employment		
Employed, *n* (%)	10 (100%)	10 (100%)
Health education teachers, *n*	1	1
Gender		
Female, *n* (%)	1 (100%)	1 (100%)
Education		
University or equivalent (>12 years), *n* (%)	1 (100%)	1 (100%)

**Table 2:** Process-evaluation components and related process measures of a complex intervention.

Process Components	Process Measures
Study population	1. Recruitment and selection rate
	2. Barriers and facilitators in recruitment and selection process
	3. Follow-up: attrition rate
	4. Barriers and facilitators for follow-up
Multiple components	1. Quality of delivery of the interventional components
	2. Barriers and facilitators for delivery of interventional components
	3. Adherence to interventional components
	4. Barriers and facilitators for adherence to interventional components
	5. Experience of participants and instructors with interventional components
Evaluation data	1. Outcome measures: coverage of interventional components
	2. Completeness of data collection
	3. Barriers and facilitators for data collection

**Table 3:** Experiences and perceptions of participating in the intervention.

Themes	Subthemes	Verbatim Quotes
Theme 1: Perceived high efficacy of the intervention components to help children become more active	(1.1) Activity diary plays an active role in helping children review behavior goals	(1.1.1) “I do try to spare some time each day reading them, and then if I find anything beneficial for me, I will just do it and if not, I will make slight adjustments.” (Child 3, Male, Intervention group).
		(1.1.2) “My favorite is the exercise diary because I can record my daily steps, so I can adjust my exercise amount according to my situation.” (Child 4, Female, Intervention group).
		(1.1.3) “The forms (daily physical activity plan and steps log) are able to motivate me to do exercise.” (Child 5, Male, Intervention group).
	(1.2) Positive impact of the group activity sessions on physical activity beliefs	(1.2.1) “The course is quite helpful. As what has mentioned in the course, physical activity is quite beneficial to our body. If you do something quite opposite to the content without even knowing it yourself, it will do harm to your body, and then it will remind yourself of how harmful it is.” (Child 1, Female, Intervention group).
		(1.2.2) “They are helpful to some extent. I know its importance. Sometimes, when I am guiding my child, parents including me will know about the importance through attending the group session.” (Parent 7, Father, Intervention group).
		(1.2.3) “Generally speaking, his participation has a positive influence on him and he is very motivated. This course is helpful for motivating his enthusiasm for exercise. He would be reluctant to record his steps if he were told to do so by the parents. However, if he were told by the teacher in the school, the result would be different.” (Parent 2, Father, Intervention group).
		(1.2.4) “It’s quite interesting. Writing the knowledge of physical activity on the handwritten poster helps one better understand such knowledge, and it is actually the self-reflection process of the knowledge.” (Child 1, Female, Intervention group).
		(1.2.5) “I am quite impressed by the last class on handwritten newspaper making…Because I think it is easier to arouse students’ interest in exercise.” (Child 9, Female, Intervention group).
	(1.3) Increased awareness of ways of doing physical activity	(1.3.1) “As long as there are suggestions for children’s guidance, we will coordinate with the arrangement of the time in and out of class. For example, we will refer to them in the spare time, and use the time after work, either at home or outside, and borrow some instructive suggestions from the materials. They are quite instructive for both us and our child.” (Parent 4, Father, Intervention group).
		(1.3.2) “Parents had no idea of them before, but they will know after reading the materials. Then parents will guide their child to do some physical activities. It is quite a good method.” (Parent 6, Father, Intervention group).
	(1.4) Increased actions of self-monitoring through the provision of pedometers	(1.4.1) “I believe that each time I had them with me, they served as a reminder of more exercise.” (Child 4, Female, Intervention group).
		(1.4.2) “The child was very happy about the pedometer given to him and liked going outside every day. In days when he was reluctant to go outside, he’d like to get some fresh air and exercise a little whenever he saw his pedometer.” (Parent 3, Mother, Intervention group).
		(1.4.3) “Sometimes, he tells me that his steps are not enough and he needs to go out for a walk. This is such a great help and also the invisible supervision.” (Parent 10, Mother, Intervention group).
		(1.4.4) “In fact, the children are quite willing to participate in this study, including the later period when they pay attention to how many steps they take every day, and even the students compete with each other, that is to say, how many steps you take every day and how many steps I take every day, and there is this kind of competition between them.” (Teacher, Female, Intervention group).
	(1.5) Positive influences of sports equipment on children’s unstructured and intermittent physical activity pattern	(1.5.1) “You gave us a pedometer further promoted our exercise in daily walking, and you gave us a skipping rope which made us actively participate in exercise such as rope skipping… The equipment provided arouses our interest in exercise and motivates us to complete the exercise.” (Child 3, Male, Intervention group).
		(1.5.2) “Sometimes they are quite convenient in use. Actually, we also have similar stuff at home and if time permits, we would love to use them.” (Parent 8, Mother, Intervention group).
Theme 2: Appreciation of the intervention features	(2.1) Understanding of intervention materials (e.g., activity diary, physical activity booklet)	(2.1.1) “I think it’s fine. Some information related to our body activity that was unknown to me has been explained.” (Child 1, Female, Intervention group).
		(2.1.2) “Everything is fine, or at least it is quite good from my non-professional point of view. All including the examples are good.” (Child 7, Father, Intervention group).
	(2.2) Satisfaction with venues	(2.2.1) “In my opinion, it will be easier to understand if the lecture is face-to-face because I can just ask you whenever it is hard to understand.” (Child 1, Female, Intervention group).
		(2.2.2) “I think it’d better not use the PE class time…You can use the time for class meetings and reading class.” (Child 4, Female, Intervention group).
		(2.2.3) “The online form is quite convenient because you can reach out to us at any time. If you choose the offline parents’ meeting, first, the time might be intense, and second, parents’ meeting is only held once in school in one semester. Therefore, the online form makes us communicate with each other at any time and it is great.” (Parent 6, Father, Intervention group).
	(2.3) Researcher’s characteristics in delivering the intervention	(2.3.1) “It is quite friendly. The vivid language you use in class makes the course very interesting.” (Child 10, Female, Intervention group).
		(2.3.2) “I believe that the communication is quite tacit.” (Parent 2, Father, Intervention group).
	(2.4) Acceptability of the group activity sessions’ duration, frequency, and timings	(2.4.1) “There is no need for any increase or decrease and such arrangement is quite appropriate for me.” (Child 3, Male, Intervention group).
		(2.4.2) “In my opinion, four classes are too much to accept and two or three classes are OK with me.” (Child 1, Female, Intervention group).
		(2.4.3) “We can increase it a little. However, we should neither increase it too much nor decrease it too much.” (Child 4, Female, Intervention group).
		(2.4.4) “I think the arrangement is quite reasonable. To my understanding, many parents have no idea of their children’s hardship. If children can attend their class for such a long time, parents can also seriously take a class that long, and I think it is OK.” (Parent 10, Mother, Intervention group).
		(2.4.5) “OK, no problem. So I need to take the class each semester. I think it will be better if it is held twice each semester.” (Parent 5, Mother, Intervention group).
		(2.4.6) “In my opinion, the time length of one class is a bit long and it can be divided into two. So it will slightly shorten the time length, am I right? It is my opinion. To be honest, it is not realistic for us to calm down and read the materials. It especially fits my situation because I have two children and am very busy. The time interval can be made shorter, that is, you can have two classes in one semester. It is my opinion because 45 min is too long for us.” (Parent 2, Father, Intervention group).
	(2.5) Social interaction and engagement	(2.5.1) “Because I want all of us to experience the feeling of exercise, enjoy the healthy and happy growth and do exercise together.” (Child 5, Male, Intervention group).
		(2.5.2) “If possible, I will recommend it…In my opinion, a lot of people have no idea what exactly physical activity is. Generally speaking, to most parents, physical activity is equal to running and walking. So we need to expand their recognition of physical activity. As a matter of fact, it is understandable that most people have such a definition of physical activity, which might be partially or incompletely correct. So everyone needs such propaganda for knowledge popularization or understanding.” (Parent 7, Father, Intervention group).
Theme 3: Factors that facilitated or impeded intervention attendance and delivery	(3.1) Gaining support from schools and education departments	(3.1.1) “In my point of view, the solution to this problem mainly depends on the school and it will be solved only if the school and the education department attach great importance to it. That’s to say, your pure dependence on parents is useless. When parents are forced to choose between study and exercise, most parents will put study ahead of exercise. As for the knowledge lectures, you need to ask the help from the education department and then require the school to reasonably allocate children’s time for study and exercise.” (Parent 8, Mother, Intervention group).
	(3.2) Provision of electronic materials	(3.2.1) “It would be better to have the electronic version of these materials. As what I said before, paper is not easy to find after a long time… There should be an official account regularly pushing information about the content or arrangement of the activity, and I think it will be great.” (Parent 6, Father, Intervention group).
		(3.2.2) “To my understanding, exercise refers to movement related to physical quality. So you may hand out some audio and video materials for the children to watch and understand what exercise is.” (Parent 9, Mother, Intervention group).
		(3.2.3) “I think it’s OK, because today’s children have a strong ability to accept, and there are many modern means of science and technology. In fact, can they provide some more such as the Internet, some examples, some good documentaries on sports and health, or some good ones? Some promotional videos and videos of knowledge can be recommended to him or the website so that children can make more use of this modern means and media means to learn more about such information at home and abroad, including paying more attention to their own health in all aspects, and even recommending some good sports methods, including what aerobics they can lead the children to do together, or I think examples can be diversified and multi-angled, so that children can actively participate in it, don’t know if it is right?” (Teacher, Female, Intervention group).
	(3.3) More physical activity options	(3.3.1) “In my opinion, exercise should not be limited to simple movements such as skipping ropes. Instead, exercise with larger intensity should be added to the program. It is OK for students to do exercise of larger intensity.” (Parent 6, Father, Intervention group).
	(3.4) More instruments for self-monitoring	(3.4.1) “I am not quite clear about it and I think it is good that each child in school is given a pedometer and it can serve as the bottom line of testing students’ possible exercise data. However, if there is such a device that can serve as functionally as the pedometer, it will be better.” (Parent 5, Mother, Intervention group).
		(3.4.2) “We may give them children some metrics or data that they can actually touch, enabling them to observe a change on a daily basis… You may provide the children with something more interesting, such as pulse measurement device, and you can have the children test other kinds of data.” (Parent 7, Father, Intervention group).

**Table 4:** Experiences and perceptions of participating in the trial.

Themes	Subthemes	Verbatim Quotes
Theme 4: Positive experiences and feelings gained through data collection process	(4.1) Understanding of the translated Mandarin version of information sheets	(4.1.1) “It is quite easy to understand.” (Child 10, Female, Intervention group).
		(4.1.2) “Well, there is no need to make any improvement and nothing is hard to understand. Everything is fine.” (Child 2, Female, Control group).
		(4.1.3) “Everything is OK and the content in it is easy to understand.” (Parent 2, Father, Intervention group).
		(4.1.4) “In fact, we are a little under-educated. So some information is hard for us to understand and some are beyond our knowledge. We can neither tell you the details nor explain ourselves well.” (Parent 9, Mother, Control group).
		(4.1.5) “No, I think it’s very good. Moreover, his informed consent form, including explanations, is particularly clear, and your affiliations are very clear at a glance, which makes it easy for everyone to have a kind of trust and be willing to cooperate. I think it’s very good.” (Teacher, Female, Intervention group).
		(4.1.6) “No. It is quite easy to understand and even the children can understand it.” (Teacher, Female, Control group).
	(4.2) Perceived “burden-free” completion of self-reported questionnaire	(4.2.1) “I felt no burden, and everything is acceptable.” (Child 3, Male, Intervention group).
		(4.2.2) “It’s quite easy to understand and there is nothing hard to understand.” (Child 6, Male, Control group).
	(4.3) Positive reflection and self-feedback gained from completing seven-day steps’ measurement	(4.3.1) “It’s quite great. I can feel my progress and my PE (physical education) score is also getting better and better.” (Child 5, Male, Intervention group).
		(4.3.2) “They were quite well organized. It’s fun to check my steps with a pedometer on me while I am walking!” (Child 2, Female, Control group).
	(4.4) Anthropometric measurement promotes self-monitoring behavior and active emotional response	(4.4.1) “In my opinion, the measurement provides me an official opportunity to understand my own weight and height. This is helpful for me to have a better understanding of myself.” (Child 1, Female, Intervention group).
		(4.4.2) “Quite comfortable.” (Child 1, Male, Control group).
	(4.5) Satisfaction regarding the organization of data collection	(4.5.1) “I am quite satisfied. It took me only a while to get measured in school before I could go back to continue my class.” (Child 1, Female, Intervention group).
		(4.5.2) “The measurement was well organized.” (Child 3, Female, Control group).
Theme 5: Satisfaction regarding the organization and implementation of the trial	(5.1) Satisfaction with the trial content, organization, and engagement	(5.1.1) “I am satisfied with most of the content and there is nothing dissatisfying about it.” (Child 2, Male, Intervention group).
		(5.1.2) “I am satisfied. The event was organized by our school and any school event does no harm but good to its students.” (Child 1, Male, control group).
		(5.1.3) “The child was very excited about it and was also willing to participate in it. He also told us about the small presents he brought back. He’d like to share everything with us. It is a good thing, I think.” (Parent 7, Mother, Intervention group).
		(5.1.4) “It’s fine. Nothing needs to be improved and everything is fine.” (Parent 7, Mother, Control group).
	(5.2) Meaningful and unprecedented experience	(5.2.1) “In my opinion, it is a new type of teaching mode, which is needed by the children. For parents, it’s something new.” (Parent 1, Mother, Intervention group).
		(5.2.2) “I hoped that it would be helpful for the child’s education.” (Parent 9, Mother, Control group).
		(5.2.3) “In fact, I think participating in this research is a very good experience for the whole students, including teachers and parents, including a very good education for us. Whether it is knowledge education or manual distribution, children are quite fresh, curious, and fresh, and I think it is a good study at the same time. This kind of knowledge education is very good for us, especially for paying attention to sports health. There are other practical components, and there are materials to distribute and then exercise. You also include the parents, I think you have considered it very carefully. I think each piece is very important and good.” (Teacher, Female, Intervention group).
		(5.2.4) “Well, first I think it is quite a meaningful activity. Under the current education circumstance, children have a limited amount of time each day for activities. Second, children may not have an adequate understanding of their own physical health. Third, I think this activity can help children develop their interest in sports in a better way. At last, this activity does good to their physical fitness and enables them to have a better understanding in all aspects.” (Teacher, Female, Control group).
Theme 6: Influences of personal beliefs and emotional responses to the trial	(6.1) Personal motivation for doing physical activity	(6.1.1) “I am a fan of sports and like running and playing football. I am also a runner and the pedometer is very useful. I’d like to know my steps.” (Child 5, Male, Control group).
		(6.1.2) “It’s great and it would be an opportunity for me to do some exercise.” (Child 1, Female, Intervention group).
		(6.1.3) “I am a teacher too, so I like participating in various activities held for children, either activities that I can understand now, or those that I cannot understand now, and we will still participate in them. Participation in various activities might have become a hobby.” (Parent 7, Father, Intervention group).
		(6.1.4) “I was quite happy when I first heard of it because it is a good thing for children to participate in physical activity. Children barely have such an opportunity to participate in such a practical study, and I think it is a rare opportunity.” (Teacher, Female, Control group).
	(6.2) Curiosity about the trial content	(6.2.1) “I participated in it out of curiosity and enthusiasm, and I did it to become healthier because physical activity makes people healthy.” (Child 5, Male, Intervention group).
		(6.2.2) “Just because I could participate in it and I never participated in such kind of activities.” (Child 10, Male, Control group).
		(6.2.3) “It was fine. As a matter of fact, I wanted to know about the specific content of this study.” (Parent 9, Mother, Intervention group).
		(6.2.4) “We wanted to know a little about our child (via participation in the trial).” (Parent 3, Mother, Control group).
	(6.3) Desire to advance physical activity knowledge	(6.3.1) “Actually, it’s because of the child. Children nowadays are lack of exercise and they like staying at home all day. I just wanted to know the child’s physical fitness and I found that his exercise amount was far less than ours when we were kids. I just wanted to participate in this activity, making us get to know what exactly physical activity is. Our previous activities are too simple.” (Parent 1, Mother, Intervention group).
		(6.3.2) “We parents want our children to be as healthy as possible and want to understand how to make the children’s body better from all aspects such as nutrition.” (Parent 6, Father, Intervention group).
		(6.3.3) “For the sake of the child’s education and for the sake of the child’s growth.” (Parent 10, Father, Control group).
		(6.3.4) “Children today have no awareness of active exercise. This activity can improve the children’s physical activity and make them less resistant to exercise.“ (Parent 2, Mother, Control group).
	(6.4) Perceived physical-activity-related benefits	(6.4.1) “I was lazy and have been stayed at home. I believe that this physical activity would make me healthier, so I wanted to know more about it.” (Child 1, Female, Intervention group).
		(6.4.2) “Well, because exercise can do some good to our body, and I’d like to give it a try.” (Child 1, Male, Control group).
		(6.4.3) “…we care more about his physical health. The learning environment nowadays has a big influence on children, resulting in less time for extracurricular activities. Therefore, I quite agree with the opportunity for exercise.” (Parent 4, Father, Intervention group).
		(6.4.4) “I am overweight but I don’t want my child to be overweight like me. He needs to start doing exercise at an early age.” (Parent 8, Father, Control group).
		(6.4.5) “…When I reported to our principal, the principal was especially willing and hoped to participate in such a study, which is also a very meaningful thing to promote the health of the whole student, so he was especially willing to participate.” (Teacher, Female, Intervention group).
		(6.4.6) “em.. in my opinion, as a teacher, the object of our education is to cultivate children as a ‘full’ person. So I think that our obligation should not just be limited to cultivating students’ academic and intellectual development, and more attention should be paid to the physical development of children, and those are the reasons.” (Teacher, Female, Control group).
	(6.5) Using vivid expressions and appealing advertising materials	(6.5.1) “If you want to arouse their interest, first, you need to use the vivid and slightly humorous language, which will make them slightly interested in it. Second, you can make some posters with interesting pictures because all my classmates like pictures and similar stuff. Posters with pictures will make them feel how great this course will be. If you want to deprive them of their interest, you just need to provide stuff in text only without any illustration, and students will soon lose patience in reading them.” (Child 10, Female, Intervention group).
Theme 7: Social influences on participatory decision-making	(7.1) Interpersonal influences by children and teachers	(7.1.1) “First, it was introduced by the headteacher. Later, I got the news. Finally, the child told us the news after school. I thought we were very lucky. We have been attaching great importance to the child. So after we received the invitation from the teacher and you, both my child and I were quite excited.” (Parent 5, Mother, Intervention group).
		(7.1.2) “I was contacted and introduced by the teacher.” (Parent 4, Mother, Control group).
	(7.2) Preferred recruitment in the school context	(7.2.1) “My personal information is safe in school and I think it’s fine.” (Child 1, Female, Intervention group).
		(7.2.2) “I am satisfied.” (Child 6, Male, Control group).
		(7.2.3) “…the only approach is through the school and teacher. You may be able to contact us personally and the school, but it seems more convenient while doing it through the school as a media, which might be easier for us to accept. In my opinion, contacting parents through the school is the most appropriate approach.” (Parent 4, Father, Intervention group).
		(7.2.4) “I’d rather not take such a risk (not having recruitment via school).” (Parent 1, Mother, Control group).
		(7.2.5) “Well, it is quite a good approach. First, I will say something about the invitation through the school. The study using such an approach is able to provide children with a guarantee in all aspects and is a relatively formal method.” (Teacher, Female, Control group).
	(7.3) Highly influenced by the curriculum that had been set up in schools	(7.3.1) “You have to make the school attach great importance to it. As long as the school pays attention to this activity, your promotion will be acceptable to students and be no problem for parents. As parents, we all wish our children have a certain amount of activities and exercise, right? Exercise is very helpful for children’s physical health. Not all energy shall be invested in the study in the classroom. As for outdoor activities, if the school can truly pay attention to them, parents will definitely cooperate with the school. As long as you can coordinate well with the school, your promotion will be quite smooth, I believe.” (Parent 4, Father, Intervention group).
Theme 8: Key barriers to consider regarding the recruitment of participants	(8.1) Academic pressure faced by children	(8.1.1) “It is possibly because my classmates have no sufficient time for participation due to study.” (Child 9, Female, Intervention group).
		(8.1.2) “A lot of people find it useless, and not mention that we have the heavy homework.” (Parent 7, Father, Intervention group).
		(8.1.3) “Some students do their homework slowly and if they are still doing the homework, they will miss this interview.” (Child 5, Male, Control group).
		(8.1.4) “Children today are lack of exercise but in the face of a lot of homework and great pressure.” (Parent 6, Mother, Control group).
	(8.2) Time constraints on parents	(8.2.1) “…First, parents do not have much time to understand your activity and they can only know it through their children in school. Parents are helpful in guidance and supervision. However, we do not have time to take the initiative to do anything.” (Parent 9, Mother, Intervention group).
		(8.2.2) “You’d better call me in the evening because I am working in the day.” (Parent 2, Mother, Control group).
	(8.3) Lack of emphasis on physical activity at parental, school, and education department levels	(8.3.1) “In my opinion, some parents will be the obstacle. Some parents put study ahead of exercise.” (Child 5, Male, Intervention group).
		(8.3.2) “Just like what I mentioned before, you need to focus on the role of the education department, that is, if the school pays attention to it, the parents will also pay attention to it. If the school ignores it, the parents will not pay much attention to it either. A lot of parents including us are following the steps of the school, that’s to say, when the school pays attention to one thing, we will also put our emphasis on it. If the school does not pay attention to it, parents will not either. It is actually a good thing. … However, pure dependence on parents is useless and you have to rely on the mutual efforts of us and the education department, as well as the participation of all parties. We have to work together and strive for the same goal. Most importantly, you have to get the attention of the education department. As long as the education department pays attention to something, parents will definitely pay attention to it.” (Parent 8, Mother, Intervention group).

## Data Availability

The data presented in this study are available on request from the corresponding author.

## Supplementary Materials

The following supporting information can be downloaded at: https://www.mdpi.com/article/10.3390/ijerph19021021/s1, Supplementary File S1: consolidated criteria for reporting qualitative studies (COREQ): 32-item checklist; Supplementary File s2: the intervention content, structure, and theoretical basis; Supplementary File S3: interview guides.
