# Supplementary material for: School-Based Physical Activity Intervention: A Qualitative Process Evaluation of a Feasibility Trial in Yangzhou, China
Source: Int J Environ Res Public Health. 2022 Jan 17;19(2):1021. doi: 10.3390/ijerph19021021 (PMC8775609; doi:10.3390/ijerph19021021)
Supplement: Supplementary file 1 [file ijerph-19-01021-s001.zip › Supplementary file 1_COREQ checklist.pdf]

**Supplementary File S1: Consolidated criteria for reporting qualitative studies (COREQ): 32-item checklist**

| No. Item | Guide questions/description | Reported on Page No. |
|---|---|---|
| **Domain 1: Research team and reflexivity** | | |
| *Personal Characteristics* | | |
| 1. Interviewer/facilitator | Which author/s conducted the interview or focus group? | Page 6 |
| 2. Credentials | What were the researcher's credentials? E.g. PhD, MD | Page 1 |
| 3. Occupation | What was their occupation at the time of the study? | Page 1 |
| 4. Gender | Was the researcher male or female? | Page 6 |
| 5. Experience and training | What experience or training did the researcher have? | Page 6 |
| *Relationship with participants* | | |
| 6. Relationship established | Was a relationship established prior to study commencement? | Page 6<br>. |
| 7. Participant knowledge of the interviewer | What did the participants know about the researcher? e.g. personal goals, reasons for doing the research | Page 6 |
| 8. Interviewer characteristics | What characteristics were reported about the interviewer/facilitator? e.g. Bias, assumptions, reasons and interests in the research topic | Page 1, 2, 3, 5, 6, 7, 22, 23 |
| **Domain 2: study design** | | |
| *Theoretical framework* | | |
| 9. Methodological orientation and Theory | What methodological orientation was stated to underpin the study? e.g. grounded theory, discourse analysis, ethnography, phenomenology, content analysis | Page 7 |
| *Participant selection* | | |
| 10. Sampling | How were participants selected? e.g. purposive, convenience, consecutive, snowball | Page 4 |
| 11. Method of approach | How were participants approached? e.g. face- | Page 4 |

| | | |
|---|---|---|
| | to-face, telephone, mail, email | |
| 12. Sample size | How many participants were in the study? | Page 4 and 5 |
| 13. Non-participation | How many people refused to participate or dropped out? Reasons? | N/A |
| *Setting* | | |
| 14. Setting of data collection | Where was the data collected? e.g. home, clinic, workplace | Page 6 |
| 15. Presence of non-participants | Was anyone else present besides the participants and researchers? | No |
| 16. Description of sample | What are the important characteristics of the sample? e.g. demographic data, date | Table 1 |
| *Data collection* | | |
| 17. Interview guide | Were questions, prompts, guides provided by the authors? Was it pilot tested? | Page 5 and 6, Supplementary file 3 |
| 18. Repeat interviews | Were repeat interviews carried out? If yes, how many? | No |
| 19. Audio/visual recording | Did the research use audio or visual recording to collect the data? | Page 6 |
| 20. Field notes | Were field notes made during and/or after the interview or focus group? | Page 6 |
| 21. Duration | What was the duration of the interviews or focus group? | Page 6 |
| 22. Data saturation | Was data saturation discussed? | Page 4 |
| 23. Transcripts returned | Were transcripts returned to participants for comment and/or correction? | Page 6 |
| **Domain 3: analysis and findings** | | |
| *Data analysis* | | |
| 24. Number of data coders | How many data coders coded the data? | Page 6 |
| 25. Description of the coding tree | Did authors provide a description of the coding tree? | Table 3 and 4 |
| 26. Derivation of themes | Were themes identified in advance or derived from the data? | Page 7 |

| | | |
|---|---|---|
| 27. Software | What software, if applicable, was used to manage the data? | Page 6 |
| 28. Participant checking | Did participants provide feedback on the findings? | Page 7 |
| *Reporting* | | |
| 29. Quotations presented | Were participant quotations presented to illustrate the themes/findings? Was each quotation identified? e.g. participant number | Table 3 and 4 |
| 30. Data and findings consistent | Was there consistency between the data presented and the findings? | Yes, there was.<br>Page 7 to 19 |
| 31. Clarity of major themes | Were major themes clearly presented in the findings? | Yes. they were.<br>From page 7 to 19 |
| 32. Clarity of minor themes | Is there a description of diverse cases or discussion of minor themes? | Discussion of major and minor themes.<br>Page 7 to 19 |
