# Supplementary material for: School-Based Physical Activity Intervention: A Qualitative Process Evaluation of a Feasibility Trial in Yangzhou, China
Source: Int J Environ Res Public Health. 2022 Jan 17;19(2):1021. doi: 10.3390/ijerph19021021 (PMC8775609; doi:10.3390/ijerph19021021)
Supplement: Supplementary file 1 [file ijerph-19-01021-s001.zip › Supplementary file 2_The intervention content, structure, and theoretical basis.pdf]

Supplementary File S2: The intervention content, structure, and theoretical basis

Intervention structure	When	Intervention contents	Intervention material	Mode of delivery	Delivered to	TDF	Intervention functions	BCTs (as numbering in BCT taxonomy)
**Health education**	Session 1 (Week 1)	a. General introduction to physical activity (definition, positives, negatives of physical inactivity).<br>b. Discussion about what sort of things made children want to do exercise and what stopped them from doing it.<br>c. Making their own weekly physical activity plan to be active at school and home for the next four weeks, including (a) what type of physical activity would you do? (b) how long? (c) how many times a week? and (d) when would you start?	a. Slide (activity plans were included in it).<br>b. Pedometer and activity diary were delivered in the session.	Face-to-face group session	Children	B con,<br>Kn	Education,<br>Persuasion	1.1 Goal setting (behavior)<br>1.4 Action planning<br>2.3 Self-monitoring of behavior<br>5.1 Information about health consequences<br>5.6 Information on emotional benefits<br>9.2 Pros and cons<br>12.5 Adding objects to the environment<br>15.1 Verbal persuasion about capability
	Session 2 (Week 5)	a. Reviewing children's physical activity plan that made in week one and gave feedback.<br>b. Providing tips for physical activity (intense, duration, safety) and motivation.<br>c. Making their own weekly physical activity plan to be active at school and home for the next four weeks, including (a) what type of physical activity would you do? (b) how long? (c) how many times a week? and (d) when would you start?	a. Slide (activity plans were included in it).<br>b. Activity diary	Face-to-face group session	Children	B con, Kn,<br>B cap	Persuasion,<br>Modelling	1.1 Goal setting (behavior)<br>1.4 Action planning<br>1.5 Review behavioral goals<br>2.3 Self-monitoring of behavior<br>4.1 Instruction on how to perform behavior<br>15.1 Verbal persuasion about capability<br>15.3 Focus on past success
	Session 3 (Week 9)	a. Reviewing children's physical activity plan that made in week one and gave feedback.<br>b. Poster making session.<br>c. Making their own weekly physical activity plan to be active at school and home for the next four weeks, including (a) what type of physical activity would you do? (b) how long? (c) how many times a week? and (d) when would you start?	Slide (activity plans were included in it).	Face-to-face group session	Children	B cap, B con	Persuasion	1.1 Goal setting (behavior)<br>1.4 Action planning<br>1.5 Review behavioral goals<br>2.3 Self-monitoring of behavior
	Session 4 (Week 13)	a. Reviewing children's physical activity plan that made in week 9 (session 3) and gave feedback.<br>b. Discussion about the poster making and ideas and then gave feedback.<br>c. Poster presentation.	Slide	Face-to-face group session	Children	B con, B cap	Enablement	1.4 Action planning<br>1.5 Review behavioral goals<br>2.3 Self-monitoring of behavior
**Family involvement**	Week 1	A physical activity session was to (a) deliver knowledge of the physical activity and tips for encouraging children to be active (including video clip demonstrations (from physical activity experts and active kids)), (b) discuss the barriers to children's physical activity and how can they help their child to be active (discussion among parents), (c) encourage parents to buddy up with the child to make activity plans (e.g. walk) and encourage/congratulate to the child if the goal has achieved.	Slide (including video clips)	Online session	Parents	SI, Kn, Id,<br>Em	Education,<br>Enablement,<br>Persuasion,<br>Modelling	1.2 Problem solving<br>1.4 Action planning<br>3.2 Social support (practical)<br>3.3 Social support (emotional)<br>4.1 Instruction on how to perform behavior<br>5.1 Information about health consequences<br>5.6 Information on emotional benefits<br>6.1 Demonstration of the behavior
	Week 1-16	Providing physical activity booklets with parents to consolidate the content of the health education.	Booklet	Physical activity booklet	Parents (distributed to parents at week one and asked them to use throughout the intervention)	SI, Kn,<br>Id	Education	4.1 Instruction on how to perform behavior<br>5.1 Information about health consequences<br>5.6 Information on emotional benefits

**School environmental support**	Week 1-16	Providing children with sports equipment to use during class break and after school.	Sports equipment	Equipment provision	Children (distributed to children at week one and asked them to use throughout the intervention)	Env, MAD	Enablement, Environmental restructuring	3.2 Social support (practical)<br>12.1 Restructuring the physical environment<br>12.5 Adding objects to the environment
	Week 1-16	Physical activity poster put in the classroom.	Poster	Equipment provision	Children (put in the classroom from week one)	Env, Goals	Environmental restructuring, Enablement, Education	7.1 Prompts/cues<br>12.5 Adding objects to the environment
	Week 1-16	Providing children with pedometers to use daily.	Pedometer	Equipment provision	Children (distributed to children at week 1 and asked them to use throughout the intervention)	Env, Goals	Enablement, Environmental restructuring	2.3 Self-monitoring of behavior<br>12.5 Adding objects to the environment
	Week 1-16	a. Providing an activity diary with each child to record their daily step count and learn activity knowledge and tips.<br>b. The activity diary includes 5-a-day physical activity concepts, which helped motivate children to participate in more physical activity and continuously use the diary. Children were rewarded with stickers if the 5-a-day physical activity task was completed.<br>c. Encouraging children to set a gradually increased goal of step counts. Children were given verbal feedback from the researcher and advice on how to achieve it.	Activity diary	Equipment provision	Children (distributed to children at week 1 and asked them to use throughout the intervention)	Env, Goals, Sk	Enablement, Training, Environmental restructuring, Education, Incentivization	1.1 Goal setting (behavior)<br>1.4 Action planning<br>1.5 Review behavior (goals)<br>2.2 Feedback on behavior<br>2.3 Self-monitoring of behavior<br>7.1 Prompts/cues<br>8.7 Graded tasks<br>10.4 Social reward<br>10.2 Material reward (behavior)

TDF domain abbreviations: Kn knowledge; MAD memory, attention and decision processes; SI social influences; Env environmental context and resources; B Cap beliefs about capabilities; B Con beliefs about consequences; Id social/professional role and identity; Em emotions; SK skills.
