# Supplementary material for: School-Based Physical Activity Intervention: A Qualitative Process Evaluation of a Feasibility Trial in Yangzhou, China
Source: Int J Environ Res Public Health. 2022 Jan 17;19(2):1021. doi: 10.3390/ijerph19021021 (PMC8775609; doi:10.3390/ijerph19021021)
Supplement: Supplementary file 1 [file ijerph-19-01021-s001.zip › Supplementary file 3_Interview guides.pdf]

Supplementary File S3: Interview guides

Interview Guide (children)

Participant ID__________

Introductions

Talk through the participant information sheet for the interview, provide an opportunity for questions about the interview and reconfirm the children's consent to take part.

Experience of taking part in the feasibility study

(1) Recruitment

1. What attracted you to taking part in this particular research study?

2. What was your initial reaction to receiving the invitation to take part in the feasibility study?

Prompts:

- Referred by the teacher, etc.

3. What did you think about the recruitment process (the way that you were recruited to the feasibility study)?

Prompts:

- What did you like/dislike about this process (e.g. referral by the teacher)? How could we improve this?

4. What did you like/dislike about the written information on the study? Whether any of it was easy or difficult to understand?

Prompts:

- Look through the participant information sheet and consent form.
- How could we improve these?

(2) Data collection

5. So far we have asked you to attend two measurement sessions. How have you found these?

Prompts:

- How have you found doing it?

- What has gone well? What has gone less well?

6. What did you think about the questionnaire booklets that we asked you to fill in?

Prompts:

- What was your feeling when you filled in the questionnaire (comfortable/burden)?

7. What did you think about the interview you have attended?

Prompts:

- What helped you to come for the final interview session? If you missed it, what would have helped you to attend? (Time/place etc)
- What did you like/dislike about the interview session?
- How might we improve the session for participants?

Experience of taking part in the intervention （intervention group only）

8. What has been your experience of attending the classroom education curriculum sessions?

Prompts:

- Did you enjoy the sessions? Did you look forward to them? What did you like/dislike about them? (Why/why not?)
- What has gone well/less well? Were they better than you anticipated? Are you satisfied with what you were offered? (Why/why not?)
- How many sessions did you attend? If you did not manage to attend the sessions, why was that?
- Was the 45-minute classroom education curriculum session once a month the right duration and frequency for you – prefer more or less? Why?
- Would you recommend it to other people?
- How can we improve the sessions? (any other ways could be used to provide information about physical activity for school-aged students?)

9. How do you think that taking part in the 15-minute discussion has had an effect on you personally (positive/negative)? Has it helped? In what ways?

Prompt:

- Physically, mentally, etc.

10. How was the overall organization of the classroom education curriculum sessions?

Prompts:

- Venue/location: Was it suitable for classroom education curriculum sessions? Was it easy to get to? Did you require any special travel arrangements?

11. What has been your experience of attending the poster-making session?

Prompts:

- Did you enjoy the session? Did you look forward to them? What did you like/dislike about them? (Why/why not?)
- What has gone well/less well? Were they better than you anticipated? Are you satisfied with what you were offered? (Why/why not?)
- If you did not manage to attend the sessions, why was that?
- Was one 45-minute poster-making session per semester the right duration and frequency for you – prefer more or less? Why?
- Would you recommend it to other people?
- How can we improve the session?

12. How was the overall organization of the poster-making session?

Prompts:

- Venue/location: Was it suitable for discussion? Was it easy to get to? Did you require any special travel arrangements?

13. How did you find the educational materials/ posted posters/messages?

Prompts:

- How useful was it in helping you encourage you to do physical activity?
- How did you use the educational materials?
- Which sections of educational materials worked well/less well? (Why/why not?)
- Was there enough support for you? (Why/why not?)
- How can we improve it?

14. Can you talk a bit about your classroom education curriculum/poster-making session instructor?

Prompt:

- Did you get on with the instructor?
- Was the instructor friendly?
- What did you think about the instructor’s approach?

15. How might we encourage more participants to come along to the classroom education curriculum/poster-making sessions?

Prompt:

- What encouraged or prevented you from attending the sessions? (Travel reimbursements etc)

16. Finally, is there anything else you want to tell us about the involved programme content?

End interview

Thank the participant and let them know that they will receive a summary of the findings of the study in due course.

Interview Guide (Parents)

Participant ID__________

Introductions

Talk through the participant information sheet for the interview, provide an opportunity for questions about the interview and reconfirm the parents' consent to take part.

Experience of taking part in the feasibility study

(1) Recruitment

1. What attracted you to taking part in this particular research study?

2. What was your initial reaction to receiving the invitation to take part in the feasibility study?

Prompts:

- Referred by the teacher, etc.

3. What did you think about the recruitment process (the way that you were recruited to the feasibility study)?

Prompts:

- What did you like/dislike about this process (e.g. referral by the teacher)? How could we improve this?

4. What did you like/dislike about the written information on the study? Whether any of it was easy or difficult to understand?

Prompts:

- Look through the participant information sheet and consent form.
- How could we improve these?

(2) Data collection

5. What did you think about the interview you have attended?

Prompts:

- What helped you to come for the final measurement session? If you missed it, what would have helped you to attend? (Time/place etc)

- What did you like/dislike about the measurement session?
- How might we improve the measurement session for participants?

Experience of taking part in the intervention （intervention group only）

6. What has been your experience of attending the educational programme?

Prompts:

- Did you enjoy the session? Did you look forward to them? What did you like/dislike about them? (Why/why not?)
- What has gone well/less well? Were they better than you anticipated? Are you satisfied with what you were offered? (Why/why not?)
- If you did not manage to attend the session, why was that?
- Have you got to know the other participants since joining the session?
- Was the 45-minute session per semester the right duration and frequency for you – prefer more or less? Why?
- Would you recommend it to other people?
- How can we improve the session? (any other ways could be used to provide information about physical activity for school-aged students?)

7. How was the overall organization of the educational programme?

Prompts:

- Venue/location: Was it suitable for discussion? Was it easy to get to? Did you require any special travel arrangements?

8. How did you find the educational materials?

Prompts:

- How useful was it in helping you encourage your children to do physical activity?
- How did you use the materials?
- Which sections worked well/less well? (Why/why not?)
- Was there enough support for you? (Why/why not?)
- How can we improve it?

9. Can you talk a bit about your programme instructor?

Prompt:

- Did you get on with the instructor?
- Was the instructor friendly?
- What did you think about the instructor's approach?

10. How do you think that taking part in the educational programme has had an effect on you and your child personally (positive/negative)? Has it helped? In what ways?

Prompt:

- Physically, mentally, etc.

11. How might we encourage more participants to come along to the educational programme?

Prompt:

- What encouraged or prevented you from attending the session? (Travel reimbursements etc)

12. Finally, is there anything else you want to tell us about the involved programme content?

End interview

Thank the participant and let them know that they will receive a summary of the findings of the study in due course.

Interview Guide (Teachers)

Participant ID__________

Introductions

Talk through the participant information sheet for the interview, provide an opportunity for questions about the interview and reconfirm the physical education teacher's consent to take part.

Experience of taking part in the study

(1) Recruitment

1. What attracted you to taking part in this particular research study?

2. What was your initial reaction to receiving the invitation to take part in the feasibility study?

Prompts:

- Referred by the school, etc.

3. What did you think about the recruitment process (the way that you were recruited to the feasibility study)?

Prompts:

- What did you like/dislike about this process (e.g. referral by the school)? How could we improve this?

4. What did you like/dislike about the written information on the study? Whether any of it was easy or difficult to understand?

Prompts:

- Look through the participant information sheet and consent form.
- How could we improve these?

(2) Data collection

5. What did you think about the interview you have attended?

Prompts:

- What helped you to come for the final measurement session? If you missed it, what would have helped you to attend? (Time/place etc)

- What did you like/dislike about the measurement session?
- How might we improve the measurement session?

6. Overall, what do you think about this study?

Prompts:

- How have you found doing it?
- What has gone well? What has gone less well?

7. How did children respond to the study?

Experience of delivering the intervention （intervention group only）

8. What has been your experience of delivering the classroom education curriculum session/poster-making session/educational programme?

Prompts:

- How have you found doing it?
- What has gone well/less well? (Why/why not?)

9. How was the overall organization of the classroom education curriculum session/poster-making session/educational programme?

Prompts:

- Venue/location: Was it suitable for the sessions? Was it easy to get to?
- Timings: physical education class or afterschool leisure time?

10. How did you find the educational materials and the programme protocol?

Prompts:

- How useful were they in helping you run the programme?
- How did you use the protocol and educational materials?
- Which sections worked well/less well? (Why/why not?)
- How can we improve them?
- Was there enough support for you? (Why/why not?)

11. How did you go about working with the children to encourage/motivate them to keep on doing physical activity?

Prompts:

- How did that go?
- What worked well/less well? (Why/why not?)

12. How did you go about working with the parents to encourage their children and to find other opportunities for activity for their children?

Prompts:

- How did that go?
- What worked well/less well?

13. Based on the response of participants, which programme component is more effective in increasing their physical activity level than the others?

Prompts:

- Why do you think so?
- Did participants seem to respond more positively to one programme content over the others?

14. How did you feel about the study components compare to the practices you usually did in your work?
15. Can you suggest other ways that could be used to provide information about physical activity for children?
16. Would you recommend this programme to other school-aged children?

Prompts:

- why yes/why not?

17. What sorts of queries children had/asked you about the behavior change intervention?

18. How do you think we could improve the behavior change intervention?

19. (In case there are few participants - why do you think so few participants attended?)

20. Finally, is there anything else you want to tell us about the behavior change intervention or the wider research study?

End interview

Thank the participant and let them know that they will receive a summary of the findings of the study in due course.
